# Chaperone Mediated Autophagy in the Crosstalk of Neurodegenerative Diseases and Metabolic Disorders

**DOI:** 10.3389/fendo.2018.00778

**Published:** 2019-01-31

**Authors:** Iván E. Alfaro, Amelina Albornoz, Alfredo Molina, José Moreno, Karina Cordero, Alfredo Criollo, Mauricio Budini

**Affiliations:** ^1^Fundación Ciencia & Vida, Santiago, Chile; ^2^Dentistry Faculty, Institute in Dentistry Sciences, University of Chile, Santiago, Chile; ^3^Autophagy Research Center (ARC), Santiago, Chile; ^4^Advanced Center for Chronic Diseases (ACCDiS), University of Chile, Santiago, Chile

**Keywords:** CMA, neurodegeneration, lipids, carbohydrates, metabolism

## Abstract

Chaperone Mediated Autophagy (CMA) is a lysosomal-dependent protein degradation pathway. At least 30% of cytosolic proteins can be degraded by this process. The two major protein players of CMA are LAMP-2A and HSC70. While LAMP-2A works as a receptor for protein substrates at the lysosomal membrane, HSC70 specifically binds protein targets and takes them for CMA degradation. Because of the broad spectrum of proteins able to be degraded by CMA, this pathway has been involved in physiological and pathological processes such as lipid and carbohydrate metabolism, and neurodegenerative diseases, respectively. Both, CMA, and the mentioned processes, are affected by aging and by inadequate nutritional habits such as a high fat diet or a high carbohydrate diet. Little is known regarding about CMA, which is considered a common regulation factor that links metabolism with neurodegenerative disorders. This review summarizes what is known about CMA, focusing on its molecular mechanism, its role in protein, lipid and carbohydrate metabolism. In addition, the review will discuss how CMA could be linked to protein, lipids and carbohydrate metabolism within neurodegenerative diseases. Furthermore, it will be discussed how aging and inadequate nutritional habits can have an impact on both CMA activity and neurodegenerative disorders.

## Introduction

### Autophagy

Autophagy is a cellular process in which proteins and organelles become degraded through lysosomes in order to maintain an adequate cellular homeostasis. In mammals, three different types of autophagy pathways have been described, (i) macroautophagy (mostly known as “autophagy”), (ii) microautophagy, and (iii) chaperone-mediated autophagy (CMA).

Macroautophagy and CMA, together with proteasome, are involved in the recycling of cellular proteins, including those that can compromise the normal physiology of the cell by adopting aberrant folded forms which are able to generate aggregates or inclusions (1). Macroautophagy is a multistep pathway that starts with the formation of an autophagosome, a double membrane vesicular structure that includes the cargo to be degraded. Autophagosome formation involves the participation of different factors like the Atg protein family, LC3-II, p62, Beclin, among others.After its formation, the autophagosome fuses to the lysosome where it releases its proteolytic material to form an autolysosome structure that finally degrades the cargo (1).

In addition to proteins, macroautophagy targets can also include damaged and/or oxidized organelles such as mitochondria (mitophagy), endoplasmic reticulum (ERphagy), peroxisomes (pexophagy), lipid droplets (lipophagy), ferritin (ferritinophagy) and zymogen granules (zymophagy) (2–5).

CMA, which will be further described below, involves the participation of HSC70 (heat shock-cognate chaperone 70 KDa) and LAMP-2A (lysosomal associated-membrane protein 2A) proteins. With respect to macro- and microautophagy, CMA has been reported only to degrade cytosolic proteins previously bound to HSC70-specific sequence (see below) (6, 7). In the case of microautophagy, it carries the degradation of proteins and organelles out, by their direct engulfment of the lysosome. In microautophagy, whereas some of the proteins can be incorporated into the lysosome through an “unspecific way”, others, like those in CMA, also need the interaction with the Hsc70 chaperone. The similarities and differences between CMA and microautophagy have recently been discussed in the review of Tekiradg and Cuervo (8).

### Chaperone Mediated Autophagy (CMA)

#### CMA Overview

Chaperone-mediated autophagy (CMA) is a specific lysosomal-dependent protein degradation pathway (9). It differs from macroautophagy because, (i) it does not involve the formation of autophagosomes and autolysosomes, (ii) its targets are cellular proteins and not organelles, (iii) the protein cargo is directly delivered into the lysosomal lumen through the interaction with HSC70 and LAMP-2A (10). HSC70 belongs to the Hsp70 protein family and it has a constitutive expression, participating principally in the CMA pathway but also in microautophagy (8). LAMP-2A protein is the A isoform of Lamp-2 (lysosome associated-membrane protein type 2) and restricts the CMA degradation process. Importantly, in CMA, selectivity resides in the fact that all CMA substrate proteins contain at least one amino acidic motif biochemically related to the penta-peptide KFERQ (11, 12). In the cell cytoplasm, HSC70 recognizes the KFERQ sequence in target proteins to form the complex HSC70-substrate. Although the temporality of this process is not well known, the complex HSC70-substrate interacts with the cytosolic tail of LAMP-2A that, in turns, drives the translocation of the target protein into the lysosome lumen (13). All cells have a basal CMA activity which helps to maintain the homeostasis of many cellular proteins. However, under certain stimulus, like nutrient starvation, serum deprivation or cellular stress (e.g., protein aggregation), CMA activity increases. This up-regulation condition can be visualized by mRNA LAMP-2A overexpression, HSC70 and LAMP-2A co-localization and LAMP-2A positive lysosomes with increased perinuclear distribution (14–16).

#### CMA Target Recognition

In 1985 Dice et al. found a pentapeptide in the Ribonuclease A (RNase A) which was necessary for the degradation of this protein by lysosomes in fibroblasts (11, 17–20). When different proteins were analyzed, it was found that the best aminoacidic sequence, to be present in a protein that is going to be degraded through CMA, corresponds to the pentapeptide KFERQ (11). The general rule, is that this sequence is composed of an amino acid Glutamine (Q), that must contain in one side (right of left) a tetrapeptide, including basic-, acidic- and hydrophobic- amino acids (11). Target proteins for CMA should contain at least one KFERQ-like domain, however some of them include more than one (21). Proteins containing a KFERQ-like domain do not enter the lysosomes by themselves, they first need to be recognized and bound by the chaperone HSC70 (22). This protein, also known as HspA8, is a 73 kDa protein belonging to the Hsp70 protein family (23). Thus, HSC70 specifically recognize the KFERQ-like motive in cytosolic proteins to target them for lysosomal degradation through CMA (22). In some cases, other co-factors, or covalent modifications have been found to be necessary for the binding between the complex HSC70-protein substrate and LAMP-2A. For example, STUB1/CHIP (a ubiquitin E3 ligase) assists the degradation of HIF1A through CMA by the ubiquitination of Lysin 63 (K63) (24) and also by supporting the interaction of LAMP-2A through its chaperon binding domain (24). In addition, covalent modifications, like acetylation and phosphorylation have been described to create KFERQ-like domains capable of targeting proteins for CMA degradation (25–29).

#### CMA Substrate Translocation and Degradation

**Figure 1:**
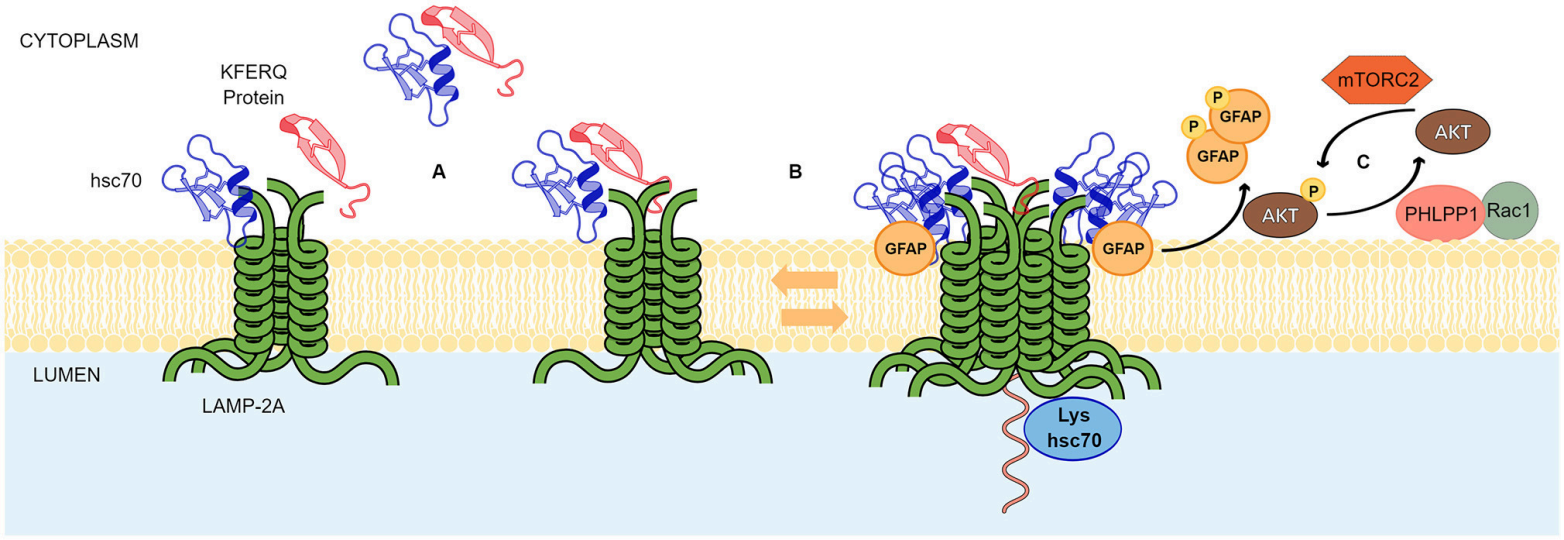
Chaperone mediated autophagy mechanism. The scheme shows the principal events in CMA mediated translocation of proteins through the lysosomal membrane and known regulatory mechanisms. **(A)** LAMP-2A exist as an inactive oligomer at the lysosomal membrane. CMA protein substrates containing KFERQ motifs and HSC70 can bind to the cytosolic tail of LAMP-2A with similar affinity. The short C-terminal tail of LAMP-2A exposed to the cytoplasm and conformed by 12 amino acids probably binds to the chaperone and the substrate in a competitive manner. However, on the substrate, binding sites for HSC70 and LAMP-2A do not overlap allowing the HSC70-substrate complexes to interact with LAMP-2A oligomer in a substrate dependent manner. **(B)** In a dynamic fashion, GFAP favors the formation of high molecular weight aggregates of LAMP2A. Somehow, coupled and simultaneous binding of the substrate to HSC70 and LAMP-2A is sensed by the transmembrane domains of the LAMP-2A for rearrangements in the supramolecular complexes. Next, substrate unfolding and translocation to the lysosomal lumen occurs. A lysosomal-HSC70 (Lys-hsc70) collaborates in this process. **(C)** A signaling pathway involving phosphorylating and dephosphorylating signals, where AKT, MTORC2, PHLPP1, RAC1, and GFAP, regulate the stabilization of the LAMP-2A complex at lysosomal membranes.

In the lysosome membrane, the HSC70-substrate complex interacts with the lysosome receptor LAMP-2A. The protein LAMP-2A was first described by Dr. Ana María Cuervo and Dr. J. Fred Dice (30). It was identified as the LGP96 (lysosome membrane glycoprotein 96 kDa) and it was found to be a necessary receptor for the specific degradation of GAPDH (Glyceraldehyde- 3-phosphate dehydrogenase) and RNase I (Ribonuclease I) through lysosomes (30). The authors showed that both GAPDH and RNase I, were able to bind the LGP96, which were isolated from rat liver (30). The Lysosome-associated membrane glycoprotein 2 (LAMP-2) is able to generate three variants through its pre-mRNA alternative splicing, (LAMP-2A, Lamp-2B, and Lamp-2C) but, to date, it is well established that only the variant A is responsible for the CMA activity (31). Compared to variants B and C, LAMP-2A has four positively charged residues in its C-terminal domain that specifically allow its interaction with target proteins (31). Characterization of LAMP-2A showed that multimerization of this variant (and not the others) in the lysosomal membrane is directly correlated with CMA activity (13, 31). The substrate up-take by the lysosome is a step that depends on LAMP-2A (32). Monomeric LAMP-2A is able to interact with substrates, however, substrate translocation is dependent on the formation of LAMP-2A oligomers (32). Further evidence showed that LAMP-2A arranges in a stable homotrimer, with helical transmembrane domains bound by a coiled-coil conformation, and with the cytosolic tails interacting with the complex HSC70-substrate protein (13). In addition, LAMP-2A oligomerization was shown to be regulated by different proteins. Glial fibrillary acidic protein (GFAP) helps to stabilize the translocation complex in an EF1α dependent manner. The stabilizing effect of GFAP is disrupted by the association of EF1α to GTP, which in turn is released from the translocation complex and allows the self-association between GFAP molecules. The self-interaction between GFAP molecules have a negative impact on the stabilization of the translocation complex. Thus, GTP acts as an inhibitor of CMA activity (33). Phosphorylating and dephosphorylating signals also regulate CMA activity at the level of the translocation complex (34). For example, mTORC2 (mammalian target of rapamycin complex 2) has been shown to activate, through phosphorylation, Akt kinase, which in turn inhibits the CMA activity. The mechanism by which active Akt decreases the CMA activity is not yet well established. However, it has been postulated that active Akt phosphorylates GFAP, destabilizing its binding to the LAMP-2A translocation complex. In this scenario, both, mTORC2 and Akt act as negative regulators of CMA activity. On the contrary, Pleckstrin homology (PH) domain and leucine-rich repeat protein phosphatase 1 (PHLPP1), has been shown to be recruited to the lysosome membrane in a Rac1 dependent manner. On the lysosomal membrane, PHLPP1 induces CMA activation by dephosphorylating Akt at the same residues previously phosphorylated by mTORC2. Thus, PHLPP1 is a positive regulator of CMA activity (34). In addition, the binding and up-take of the CMA substrates through the translocation complex is assisted by HSC70 and Hsp90 (heat shock protein 90) chaperones. In the case of Hsp90, it was shown that this protein stabilizes the binding of LAMP-2A at the lysosomal membrane (32). HSC70, apart from its important role in binding to the KFERQ domain, participates in two additional steps of the CMA process. One step is related to the recycling of LAMP-2A in the absence of substrates, where HSC70 has been proposed to support the destabilization of LAMP-2A from the translocation complex (32). The other step occurs at the lumen of the lysosome, where a lysosomal HSC70 (lys-HSC70) is also involved in the up-take process of the substrate. The blockage of lys-HSC70, by a specific antibody, has been directly correlated with an inhibition of the CMA activity (35). Figure 1 summarizes the main aspects of the CMA mechanism.

#### CMA Activation

Koga et al. (36) showed that basal CMA activity is present in a variety of cell types. However, an up-regulation in this pathway can be observed under different stimulus or conditions. The most common stimulus for CMA activation is nutrient deprivation (or starvation). Starvation activates CMA both *in vitro* and *in vivo* (11, 20, 36) and, although the exact mechanism has not been described yet, at least *in vivo*, it has been proposed that it depends on the circulating ketone bodies (37). CMA over-activation has also been observed under DNA damage. Under this condition, CMA activity is up-regulated with the purpose to degrade the checkpoint protein kinase 1 (Chk1). The accelerated degradation of Chk1 by CMA reduces its nuclear entrance and consequently decreases the phosphorylation and destabilization of the MRN (Mre11–Rad50–Nbs1), a complex that participates in the early steps of particular DNA repair pathways (38). In line with this result, DNA irradiation of a hepatocellular carcinoma cell line also over-activates CMA. In this cell line, CMA up-regulation was responsible for the degradation of HMGB1 (high-mobility group box 1 protein) degradation, which in turn, provoked the down-regulation of the p53 protein and the consequent decrease in the apoptotic rate. In this case, CMA activation could be considered a mechanism by which this type of carcinoma is able to resist the irradiation treatment (39). Oxidative stress also activates CMA constitutively. Both, mRNA LAMP-2A levels and the recruitment of LAMP-2A protein to the lysosomal membrane (40, 41) were augmented under oxidative stress. The reasons by which CMA is activated under oxidative stress are unknown. However, a possibility could be that CMA acts as a degradative backup system for an inhibited macroautophagy pathway (42). With respect to the molecular mechanism, recent work showed that under hydrogen peroxide treatment, the transcription factor NFE2L2/NRF2 (nuclear factor, erythroid derived 2, like 2) can drive the expression of the lamp2 gene. Pharmacological activation of NFE2L2/NRF2 further supported the role of this transcription factor on lamp-2A gene overexpression (43). However, under oxidative stress, CMA up-regulation could also have a toxic effect. It was shown that CMA over-activity strongly reduces the levels of MEF2A (myocyte enhancer factor 2a), a protein necessary for the adaptive response of the cells to oxidative stress (41). Another stressor that up-regulates CMA activity is hypoxia. Over-activation of CMA was directly correlated with the survival of neuronal cells exposed to different hypoxic conditions and, on the contrary, the down-regulation of this pathway sensitized the cells to the stress (44). On the other hand, it was reported that HIF-1α, a protein that activates HIF-1 (Hypoxia-inducible factor-1) and mediates an adaptive response to hypoxia, is degraded by CMA in cells exposed to hypoxic conditions (45). Therefore, the exact mechanisms by which CMA protects cells against hypoxia, needs to be further elucidated. A constitutive activation of CMA has also been observed in different models of neurodegenerative diseases. For example, in cellular and mouse models of Huntington Disease, CMA was activated as a compensatory mechanism for the inhibition of macroautophagy. In this case, both lys-HSC70 and LAMP-2A protein levels were markedly augmented. In particular, there was an observed stability in LAMP-2A proteins at the lysosomal membrane and a transcriptional upregulation of this splice variant (46). Up-regulation of CMA was also observed in molecular mechanisms related with Parkinson's disease. For example, recent studies performed with a dopaminergic neuronal cell line, using an endoplasmic reticulum (ER) stress mouse model, showed that ER stressors are able to induce CMA activation. The mechanism for such activation involved the recruitment of MKK4 protein kinase to the lysosomal membrane and the subsequent phosphorylation of LAMP-2A by p38 MAPK protein kinase (47). This regulatory mechanism for CMA up-regulation is relevant as neurotoxins associated with PD are directly correlated with an activation of the ER-p38 MAPK–CMA response pathway (48). Additionally, in PD models, LAMP-2A and HSC70 were observed to be up-regulated when α-syn was over-expressed *in vivo*, indicating that a CMA over-activation is triggered in response to a α-syn pathological condition (49)

### CMA and Metabolism

#### Participation of CMA in the Regulation of General Protein Metabolism

Protein homeostasis is regulated by a series of interconnected signaling pathways that sense amino acid availability, among them, the integrated signaling pathway (ISR) through the general control nonderepressible-2 protein (GCN2) and the V-ATPase/Ragulator/mTOR pathway located in lysosomes (50, 51). GCN2 is a protein kinase that is activated in the presence of accumulated deacetylated tRNAs, sensing amino acid depletion. The main target of GCN2 is the eukaryotic initiation factor 2 (eIF2), whose phosphorylation induces the inhibition of general translation and favors the synthesis of a specific set of mRNAs that regulates cell survival and promotion of macroautophagy (52). On the other hand, mTORC1 kinase is activated in response to amino acid availability, promoting translation and inhibiting macroautophagy. The mechanism of amino acid sensing, coupled with mTOR activation, involves the lysosomal amino acid transporter SLC38A9, and mTOR-binding proteins, Sestrin2 and Castor1 (50). These factors that regulate mTOR activation or inhibition, also modulate mTOR activity and thus mTOR-downstream targets involved in promotion of protein translation, cell growth and anabolism, and inhibition of macroautophagy initiation. In response to amino acid deprivation, mTOR activity is attenuated, protein translation is inhibited and macroautophagy is maximally activated to restore amino acid levels.

These non-selective mechanisms of protein homeostasis, added to a variety of mechanisms of stabilization and destabilization of protein pools, define the average life time of every protein in the cell, in nutrient rich conditions and in response to nutritional stress. Selective mechanisms of protein degradation are in contrast, generally linked to the modulation of the function of specific proteins or to the degradation of misfolded proteins that have been targeted for efficient elimination. Selective and non-selective mechanisms of proteasomal and macroautophagy protein degradation pathways have been extensively reviewed in several articles (53–56). Whereas proteins degraded by CMA need to be recognized specifically by HSC70 protein through its KFERQ domain, CMA can be considered a selective protein degradation process. An initial analysis indicated that at least 30% of cytosolic proteins contain CMA KFERQ-like motifs (7). These proteins mainly correspond to a fraction of long-lived proteins that were more rapidly degraded by lysosomes in response to serum starvation or amino acids withdrawal. Indeed, at least 90% of enhanced proteolysis observed during serum withdrawal in fibroblast cells is thought to be part of the protein pools targeted for degradation by CMA (20). In comparison to macroautophagy, the increase in degradation rates of the pool of proteins targeted to CMA is observed in response to prolonged periods of serum or nutrient deprivation (7, 12, 57). Indeed, macroautophagy activity decreases in activity after 6–12 h of nutrient removal, but CMA progressively increases in response to prolonged periods of serum or nutrient deprivation (57). Thus, CMA is a protein degradation pathway specifically activated during long term starvation. The pool of proteins degraded by CMA is very specific and different to the protein pools degraded by other protein catabolic neutral or acid proteolytic pathways. Additionally, CMA-targeted proteins accumulate in lysosomal membranes under starvation and, probably, because of this reason, this pool is excluded for degradation by proteasome or macroautophagy.

The pool of proteins targeted by CMA depends, most likely, on the periods of deprivation, type of deprivation (glucose, amino acids, serum or specific growth factors) and the cell type in the study (12). In addition, the selective pool of proteins degraded by CMA would also be associated with certain functions capable of overcoming metabolic changes in response to starvation. This has been observed in the regulation of glycolytic flux, through the degradation of glycolytic enzymes by CMA, in order to protect cells from apoptosis (7, 21). On the other hand, proteins that do not contain KFERQ-like regions would most likely be protected against degradation, to sustain the critical functions of cells under nutritional stress. It has also been suggested that activation of non-selective macroautophagy in the first stages of starvation, help to provide metabolite building blocks (as amino acids) for the continuous synthesis of macromolecules. However, at prolonged times of starvation, a relief of macroautophagy by selective CMA-dependent protein degradation, would be necessary to avoid the elimination of essential proteins for cell survival (57). It is unknown whether nutrient deprivation directly controls the CMA activity, as with macroautophagy. The main hypothesis is that an indirect regulation of CMA by nutrient deprivation would be carried out by translational and transcriptional up-regulation of lysosomal proteins and genes involved in lysosome biogenesis (58–60). However, a recent report indicates that upregulation of CMA was responsible for mTOR activation by a mechanism that sensed the augmentation of the free amino acid content dependent on CMA activation (61). In addition, increased CMA activity, in response to LAMP2-A overexpression, was also shown to down-regulate macroautophagy through mTOR activation (62). Overall, this latter evidence reveals that CMA activation functions as a negative feedback loop for mTOR and, thus, would regulate the general amino acid and protein homeostasis signaling.

As mentioned before, basal CMA activity can be detected in different tissues, suggesting that this pathway has a constant role in the regulation of certain proteins, even under conditions of nutrient or growth factor availability (57). However, whether the degradation of these target proteins increases under CMA activation (e.g., starvation), or another specific cell response, remains to be clarified. Most likely, the latter will depend on the different affinities that these proteins would have for HSC70 and LAMP-2A, and/or on the availability of HSC70 and LAMP-2A (63).

As a consequence of its important role in controlling protein homeostasis, CMA is able to regulate metabolic pathways that are crucial to maintain balance in cellular and systemic physiology.

#### CMA and Regulation of Lipid Metabolism

Neutral cytosolic lipolysis and lysosome-associated acid lipolysis were classically classified in two different pathways. However, both are now recognized as synergistic, cooperative and interconnected mechanisms that contribute to lipid catabolism (64). Lysosomal lipolysis involves the degradation of lipids derived from endocytosis, as well as from cytosolic lipid stores (lipid droplets) through an autophagic process known as Lipophagy (65). Lipid droplets (LDs) are specialized organelles that store neutral lipids during fatty acid availability, mainly triglycerides (TG) and cholesterol esters, for their posterior use as energy or precursors for lipids and steroids (66). LDs consist of a core of neutral lipids separated from the aqueous cytoplasm by a phospholipid monolayer decorated by a set of proteins. These proteins are enzymes involved in lipid synthesis and lipid hydrolysis, membrane-trafficking proteins and a set of specialized LDs-associated structural proteins called perilipins (PLINs). PLINs cover a family of 5 major proteins and some additional splice variants expressed in lower amounts. Perilipin 1 (PLIN1) and 2 (PLIN2) are exclusively associated with LDs whereas perilipins 3–5 are found in the cytoplasm, associated with LDs, as well as microdroplets and lipoprotein particles (67). Perilipins 1, 2 and 5 control, at the surface of the lipid droplets, the access of cytosolic neutral lipases. For example, phosphorylation of PLIN1 and PLIN5 by protein kinase A (PKA), in response to hormones or growth factors, trigger the recruitment of neutral lipases like hormone sensitive lipase (HSL) and triacyl-glycerol lipase (ATGL) (68, 69). In contrast to PLN1 and PLIN5, PLN2 is not phosphorylated by PKA and does not recruit lipases at LDs (67). Similar to PLIN2, PLIN3 and PLIN4 protect LDs from degradation and they seem to have a tissue-specific function in the regulation of LDs size, maturation and mitochondrial association (70–72).

The involvement of autophagy in lipid turnover was first demonstrated for macroautophagy (65). Electron-microscopy studies indicated that autophagosomes engulf LDs or small portions of large LDs for degradation, forming autolipophagosomes in a process now known as macrolipophagy. In hepatocytes, macrolipophagy and the presence of lipid-containing autophagolysosomes, is stimulated by nutrient starvation and low fatty acid treatments (65). Members of the Rab GTPase family, RAB7 and RAB10 have been involved in the early steps of autophagosome recruitment to LDs (73). Moreover, the Rab effectors, EHBP1 and EH2, have been suggested to help in the elongation of autophagosomal membranes around LDs (74, 75). Neutral lipases ATGL and HSL contain LIR motifs (LC3-interaction region) and interact with the cytosolic face of autophagosomes through LC3-II, being recruited to LDs in response to macrolipophagy activation (64, 76).

In addition to macroautophagy, CMA also plays a central role in lipid homeostasis. In a mouse model, where LAMP-2A was specifically down-regulated in liver by deleting the exon 9 of lamp2 gene, it was shown that the constitutive blockade of CMA activity induces pronounced steatosis without a substantial increase in TG synthesis (77). In addition, LAMP-2A knock-out cells were insensitive to lipolysis induced by starvation and displayed increased density, size and occupancy area of LDs (78). Interestingly, in this model, non-selective macroautophagy was intact, indicating a specific and new role of CMA in lipophagy (77). In line with this evidence, further studies found the presence of KFERQ-like motifs in PLIN2 and PLIN3 proteins, and identified these two proteins as substrates for CMA lysosomal degradation. In addition, lysosomes actives in CMA isolated from rat liver, were enriched in PLIN2 and PLIN3, supporting the fact that these proteins are CMA substrates. This evidence was further complemented by experiments indicating that PLIN2 and PLIN3 degradation was reduced in LAMP-2A knockout cells (78). With regards to the mechanism, the degradation of PLIN2 through CMA in response to nutrient starvation and lipid challenges, was correlated with an increment in the association between HSC70 and perilipins (78). Reduced degradation of PLIN2 in cells that are deficient for the CMA pathway, leads to reduced lipolysis, likely due to an impairment in the association of ATGL with LDs as well as due to the defective recruitment of autophagosomal proteins and Rab7 to LDs (25, 78). Moreover, PLIN2 phosphorylation by AMPK was found to be a necessary modification for PLIN2 association with HSC70 in a form dependent on CMA activation (25, 78). Therefore, the role of CMA in lipophagy not only relays in the regulation of the recruitment of neutral lipases to LDs trough perlipin degradation, but also in the integration of a regulatory step that involves a post-transcriptional modification guided by AMPK (25). Thus, CMA itself could be considered a nutrient sensing pathway supporting the fact that lysosomes can play a role as initial sensors and integrators of lipid homeostasis (79). Additional research, however, is needed to determine how cellular pathways involved in cellular lipid homeostasis communicate with CMA and lipophagy, and how CMA-dependent lipolysis affects different tissues.

#### CMA and Regulation of Carbohydrate Metabolism

As mentioned before, glycolytic enzyme glyceraldehyde 3-phosphate dehydrogenase (GAPDH) was one of the first characterized as a CMA substrate (80). Additionally, at least 8 of 10 glycolytic enzymes contain CMA-targeting motifs (77). Substrates of CMA that participate in glycolysis, validated by LAMP2-A loss of function or lysosome-transport assays, also include hexokinase-2 (HK-2) (28), the M2 isoform of pyruvate kinase (81), aldolase-A, enolase-1, 3-phosphoglyceric phosphokinase, phosphoglycerate mutase, glucose-6-phosphate-dehydrogenase and the mitochondrial proteins glutamate dehydrogenase, ornithine transcarbamoylase and malate dehydrogenase (77). Other related substrates that have been suggested to be regulated by CMA in their function include phosphofructokinase-2 (82) and aldolase B (83). Glycolytic flux regulation by CMA has been confirmed *in vivo* in a mouse model with specific down-regulation of LAMP-2A in hepatocytes (77). These mice displayed higher protein levels of glycolytic enzymes and enzymes from the tricarboxylic acid cycle (TCA), a reduction in hepatic gluconeogenesis, lower glycogen synthesis and an increase in lactate production and TCA intermediates (77). This metabolic profile suggests a switch in hepatic metabolism to carbohydrate consumption as a source of energy vs. glucose biosynthesis in response to low CMA activity (77). On the other hand, classical inhibition of hepatic glycolysis caused by serum starvation (84) was not observed in mice with liver-specific CMA down-regulation (77). These results suggest that CMA activity would be necessary for a metabolic adaptive mechanism that triggers glucose production in liver to support peripheral organs under nutritional stress conditions.

The mechanism regulating CMA in response to changes in glucose availability are not fully understood. Pointing to a central role of the lysosome in sensing glucose homeostasis, new evidence indicates that glucose starvation induces changes in lysosomal acidification in an AMPK activity dependent-manner (84, 85). The mechanisms implicated in this regulation may involve a glucose-dependent regulation of the lysosome biogenesis through the transcription factor EB (TFEB) (86). Additional research is needed to elucidate how these lysosomal changes, induced by carbohydrate availability, regulate the CMA activity and, in turn, how this affects the cellular glycolytic flux.

### CMA and Neurodegenerative Diseases

There is increasing evidence supporting the idea that dysregulation in the CMA pathway plays a crucial role in neurodegeneration.

#### Parkinson's Disease (PD)

Evidence indicates that a dysregulation in CMA could impact on the onset or progression of Parkinson's Disease (PD). As mentioned above, the main protein associated with this neurogenerative disorder, alpha-synuclein protein (α-syn), has been identified as a CMA substrate (87). More specifically, reduced α-syn degradation was observed when its KFERQ motif was mutated and the expression of LAMP-2A was knocked-down. The involvement of CMA in α-syn degradation was confirmed in different neuronal cell lines (PC12 and SH-SY5Y) and primary cultures of cortical and midbrain neurons (87). One of the hallmarks of PD is the neurotoxicity caused by the abnormal aggregation of α-syn. In this context, mutations in the protein impair its degradation through a CMA pathway, causing the accumulation α-syn oligomers that are unable to be degraded by the lysosome. This event blocks the entire CMA pathway, enhancing the oligomers formation and compromising the degradation of other CMA substrates (88, 89). As mentioned above, LAMP-2A and HSC70 were observed to be up-regulated when α-syn was over-expressed *in vivo* (49). In line with these results, it was shown that the down-regulation of LAMP-2A in adult rat substantia nigra, via an adeno-associated virus, induced intracellular accumulation of α-syn puncta. In addition, LAMP-2A down-regulation was also correlated with a progressive loss of dopaminergic neurons, severe reduction in striatal dopamine levels/terminals, increased astro- and microgliosis and relevant motor deficits (90). Furthermore, studies using the *Drosophila melanogaster* model, showed that the overexpression of human LAMP-2A protein protected the flies from progressive locomotor and oxidative defects induced by neuronal expression of a human pathological form of α-syn (91). Also, other PD related proteins seem to be regulated by CMA activity. This is the case of PARK7/DJ-1 and MEF2D. PARK7/DJ-1 is an autosomal recessive familial PD gene that plays a critical role in the antioxidative response and its dysfunction leads to mitochondrial defects. CMA was shown to degrade, preferentially, an oxidized and altered form of PARK7, providing protection from mitochondrial damage and impairing cell viability (92). For MEF2D (myocyte-specific enhancer factor 2D protein), it was shown that an inhibition of CMA provoked the accumulation of this protein in the cell cytoplasm (93). These results are in line with the observation that MEF2D levels are increased in brains of α-syn transgenic mice and in samples from patients with PD (93). Thus, an impairment in CMA activity, caused by the α-syn oligomers, could be the reason for MEF2D accumulation in PD. Beyond the studies performed in cell cultures and *in vivo* models, strong evidence indicates that CMA could also be dysregulated in individuals affected by PD. In this regard, LAMP-2A and HSC70 were observed to be down-regulated in PD patients, suggesting that a CMA dysregulation can occur before the appearance of α-syn aggregation and other PD associated disorders (94, 95). Although the mechanisms that lead to a reduction in LAMP-2A and HSC70 levels in PD remain unknown, some studies suggest that genetic variations in the promoter of the lamp-2A gene and the up-regulation of different microRNAs that target both LAMP-2A and HSC70, could be implicated in their down-regulation (96, 97).

#### Alzheimer Disease (AD)

The association between CMA and Alzheimer's Disease (AD) can be described by the degradation of the RCAN1 protein (Regulator of calcineurin 1) through this pathway. Two KFERQ-like motifs identified in RCAN1 were responsible for its degradation by the CMA pathway (98). In addition, the inhibition of CMA pathway increased RCAN1 protein levels and consequently reduced the NFAT transcription factor activity (98). Interestingly, NFAT has been involved in the transcriptional regulation of the lamp2 gene (99). Thus, inhibition of CMA activity could be further enhanced by increased levels of RCAN1, that will subsequently impair NFAT-dependent transcription of lamp2. On the other hand, the Tau protein, one of the principal factors associated with AD, has also shown to be a CMA substrate (100, 101). The Tau protein is degraded by the autophagy–lysosomal system producing different fragments that, in turn, bind to HSC70 and become CMA substrates. Although these fragments are able to reach the lysosomes, they remain bound to the lysosomal membrane, causing the formation of pathological Tau aggregates that cause lysosomal damage and block the degradation of other CMA targets (100). Additionally, the amyloid precursor protein (APP), can be processed to produce another pathogenic molecule associated with AD, the β-amyloid peptide (Aβ), which contains a KFERQ related motif (102). However, deletion of KFERQ did not affect its binding to HSC70, but did somehow keep the APP and its C-terminal fragments (CTFs) away from lysosomes. Thus, the KFERQ-like domain of APP is relevant to preclude the accumulation of APP and its CTFs but, in this case, in an CMA degradation independent manner (102).

#### Huntington Disease (HD)

As previously mentioned, a constitutive activation of CMA has been observed in cell lines and mouse models of HD (46, 103). In a mouse model for this disease, a strong co-localization between LAMP-2A and HSC70 was correlated with augmented lysosomal degradation of the native and aberrant huntingtin protein (Htt) (46). Furthermore, additional studies performed *in vivo* also showed that Htt aggregates could be forced to be degraded through CMA, by targeting them with a polyQ binding protein (QBP1) including a KFERQ domain in its amino acidic sequence (104). The data indicated that modulation of the CMA pathway can be a plausible strategy for HD treatment.

#### Amyotrophic Lateral Sclerosis (ALS) and Frontotemporal Lobar Degeneration (FTLD)

Amyotrophic Lateral Sclerosis (ALS) and Frontotemporal Lobar Degeneration (FTLD) are two important neurodegenerative diseases where the implication of CMA has been poorly studied. So far, only two studies have connected these disorders with CMA and both focus on the ribonuclear protein TDP-43. Huang et al. described an KFERQ-like domain in TDP-43 that was responsible for the interaction of this protein with HSC70 under ubiquitination condition. Mutation of the KFERQ-like domain disrupted the ubiquitin-dependent binding of TDP-43 with HSC70. In addition, the down-regulation LAMP-2A by siRNA treatment, seems to increase the level of the pathologically-related 25-KDa and 35-KDa TDP-43 C-terminal fragments, but not the full-length protein (105). On the other hand, in a recent study, Tamaki, Y., *et al* designed an antibody able to recognize abnormal (unfolded or mislocated) TDP-43. The addition of the KFERQ sequence to this antibody, was able to promote the degradation of abnormal TDP-43 through the CMA pathway. Although in this case the connection of TDP-43 with CMA was not in a physiological context, this work indicates that a forced CMA target degradation of TDP-43 can be used as a strategy to ameliorate neurodegenerative diseases associated with this protein (106).

### CMA in the Crosstalk With Metabolic Disorders and Neurodegeneration

#### Reduced CMA Activity With Aging

Aging can be considered as a physiological process that is associated with metabolic and neurodegenerative disorders via mechanisms that in many cases are completely unknown. With regards to autophagy, it has been observed that this lysosomal degradative mechanism also declines with age (107). In the case of CMA, this was determined by isolating lysosomes from the liver of young and old rats (108). Lysosomes from old rats had reduced LAMP-2A protein levels at both, total and lysosomal levels. The decrease of the LAMP-2A protein in lysosomes is associated with changes in lipid composition of these organelles that accelerate the degradation rate of the protein (108). With respect to the HSC70 protein, although the total protein levels remained unchanged, lysosomal HSC70 levels were augmented, probably to compensate the decline observed in the CMA activity (108). Further experiments demonstrated that, although the levels of Lamp2A mRNA were unchanged, there was a problem in the localization of LAMP-2A at the lysosomal membrane in old rats, consequently reducing the CMA activity (109). Furthermore, by using an animal model with specific down-regulation of CMA in the liver, it was possible to demonstrate that reduced CMA activity in old animals had an impact on the ability to overcome proteostasis induced by different stressors like oxidative stress, lipid challenging and aging (110). The mentioned evidences clearly demonstrate that CMA activity is reduced in older individuals and that this protein degradative pathway helps to stabilize the different disorders associated with aging.

Thus, considering that CMA activity is reduced with age, it is possible to argue that a decline in CMA activity during aging can be a risk factor for the development of neurodegenerative disorders associated with adult and senior people. In fact, beyond of what it can be concluded from *in vitro* and *in vivo* models, more evidence is coming out revealing that a dysregulation in CMA activity can be present in human neurodegeneration. Most of the evidence is a result of studies on PD. For example, a study performed with peripheral blood mononuclear cells from PD patients, showed that affected people had reduced HSC70 protein levels (111). Another study, analyzing CSF (cerebrospinal fluid) of PD patients with mutations in the repeat kinase 2 (LRRK2) gene, confirmed that LAMP-2A protein levels were reduced in affected females, compared to healthy people (112). However, studies focused on analyzing the levels of LAMP-2A and HSC70 in healthy people during normal aging, failed to find any positive association (113). Thus, future studies need to focus on whether changes in CMA activity (or CMA protein players) are altered during normal aging in healthy people, or if they are only associated with patients affected by neurodegeneration. The latter will help to elucidate whether observed changes in CMA protein players are a cause or a consequence of these disorders.

#### CMA Activity, Metabolic Disorders and Neurodegenerative Diseases

*In vivo* models strongly support the hypothesis that CMA activity decreases with aging. Considering this evidence, which of these could be the consequence of CMA activity in people with a high fat diet (HFD)? Rodriguez-Navarro et al. showed that chronic exposure of rats to an HFD or cholesterol-enriched diet, provoked a decrease in CMA activity (114). Further characterization of these animals showed that isolated lysosomes from liver have reduced LAMP-2A protein at lysosomal membranes. The latter was triggered by an accelerated degradation of lysosomal LAMP-2A, something also observed in older animals. Interestingly, authors also found that lysosomal membranes from animals with a HFD presented changes in lipid composition, in a similar way than that was observed with age (114). Thus, a dysregulation of CMA activity produced by aging, could impact on the LDs composition, affecting the aggregation of some proteins involved in neurodegenerative diseases.

In addition, a systemic decrease in CMA activity, and the onset or progression of neurodegenerative disorders, could be enhanced by an HFD. Different works demonstrate that a HFD can be a risk factor for the development of neurodegenerative diseases. For example, in a mouse model of PD, it was observed that HFD provoked a reduction in Parkin protein levels (115). In addition, similar results were obtained with a α-syn transgenic mouse model of PD. Compared with wild type mice, an HFD causes obesity and glucose intolerance in transgenic mice. Furthermore, transgenic mice also had an accelerated onset of PD disease and premature movement phenotype and death (116). The same situation was observed in transgenic mice models for AD. The model with an HFD, presented exacerbated neuropathology, defects in synaptic stability/plasticity, apoptotic neuronal cell death (117) and increased levels of insoluble amyloid-β (AB) and Tau (118). On the other hand, LDs have been found to play a role in neurodegenerative diseases. For example, as reviewed by Pennetta et al. LDs seem to be important for the progression of motor neuron diseases (MND) (119). The mechanisms by which LDs are affecting neurodegeneration processes are unknown, however it has been observed that they can affect protein aggregation. For example, in cells treated with an HFD, α-syn accumulated in triglyceride-riche LDs (120). In a similar way, amyloidal fibrils from amyloidotic polyneuropathy (FAP), were observed to be colocalizing with a high density lipoprotein (HDL) (121). Altogether, these data support the idea that LDs could be involved with the aggregation process of neurodegenerative disease associated proteins.

On the other hand, there is increasing evidence which now supports the idea that a high carbohydrate diet (HCD) is a risk factor for certain neurodegenerative diseases (122). Probably, because of a dysregulation in protein glycation, which can control the balance between protein solubility and aggregation (123, 124). Moreover, many reports indicate that there is a strong association between diabetes and people affected by AD (125, 126), suggesting that a dysregulation in glucose metabolism could impact on the onset of this and other neurodegenerative diseases (127). Although the mechanisms are not clear, evidence has shown that there is an increase in the activity of glycolytic enzymes in AD patients (128). In addition, α-syn was observed to bind GAPDH, increasing the activity of this enzyme (129). Also, analyses performed with postmortem human brain tissue, reported a reduction of glucose-6-phosphate dehydrogenase and 6-phosphogluconate dehydrogenase in AD and PD (127).

**Figure 2:**
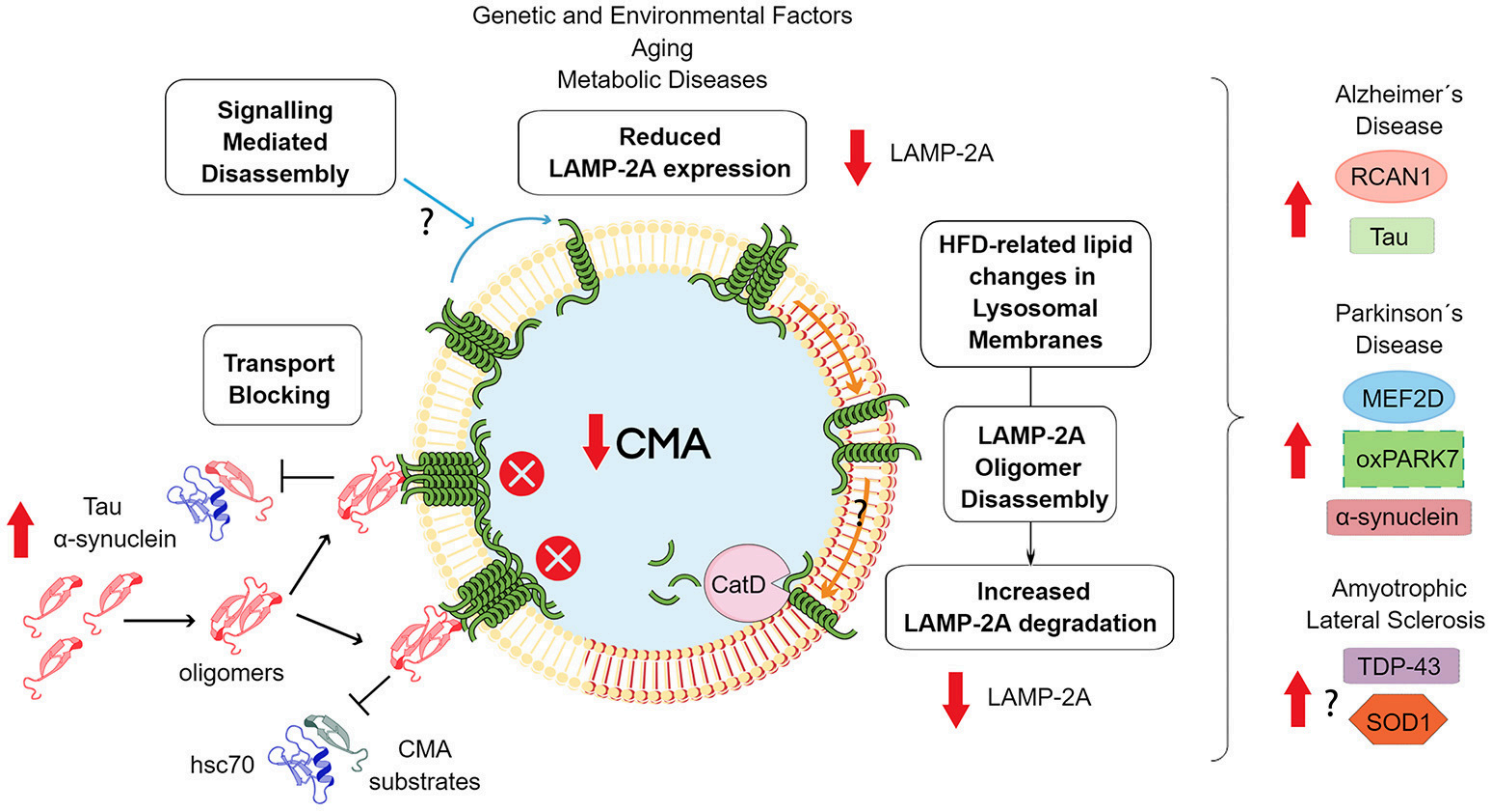
Consequences of downregulated CMA activity. The scheme shows the principal mechanisms of CMA downregulation. Reduced expression of the lamp2 gene has been related with genetic polymorphisms (96), aging (108) and metabolic disorders (132). In relation to the latter situation, HFD has been shown to modify the lipid composition of lysosomal membranes, including increased levels of cholesterol and ceramide (132). These modifications induces the formation of organized lipid microdomains, which prevent LAMP-2A multimerization and stimulate subsequent degradation through proteolysis by Cathepsin D (CatD) (133). Independently of the mechanism, a reduction in CMA activity can induce an increase in the levels of some proteins related with the development of neurodegenerative diseases. In the case of tau, α-synuclein or other less characterized CMA protein substrates that are able to aggregate, increases the total mass that these proteins would contribute to protein oligomerization, an event associated with a blockade of CMA transport. In addition, oxidative damage, would enhance the aggregation process. Additionally, changes in signaling pathways that control LAMP2A oligomerization and membrane stabilization can alter LAMP-2A membrane dynamics and CMA activity. Finally, CMA down-regulation can alter a series of other specific proteins related with neuronal survival or neuroinflammation, that participate in the development of neurodegenerative diseases.

Carbohydrate metabolism dysregulation has also been found in different models of HD. Gouarné et al. found that a rat model of HD had a deficit in glycolysis in striatal neurons (130). In a mouse model of HD, it was observed that glycolysis inhibition decreases the levels of glutamate transport and provoked neurotoxicity (131). On the contrary, in two different models of HD, it was found that the activity of some glycolytic enzymes was higher. The latter results were not correlated with the enhanced conversion of glucose to lactate and increased ATP in the brain and tissue, respectively. Altogether, this evidence indicates that in neurodegenerative diseases, an imbalance in a glycolytic pathway could play an important role in the onset or progression of the disease. Considering that CMA regulates the homeostasis of glycolytic enzymes, changes in the activity of this pathway along with aging could strongly impact on the risk to develop a neurodegenerative disease. In addition, the risk can also be augmented by aging and by a continuous intake of a high carbohydrate diet. Figure 2 shows the main mechanisms that can influence CMA down-regulation and its impact in neurodegenerative diseases.

## Concluding Remarks

**Figure 3:**
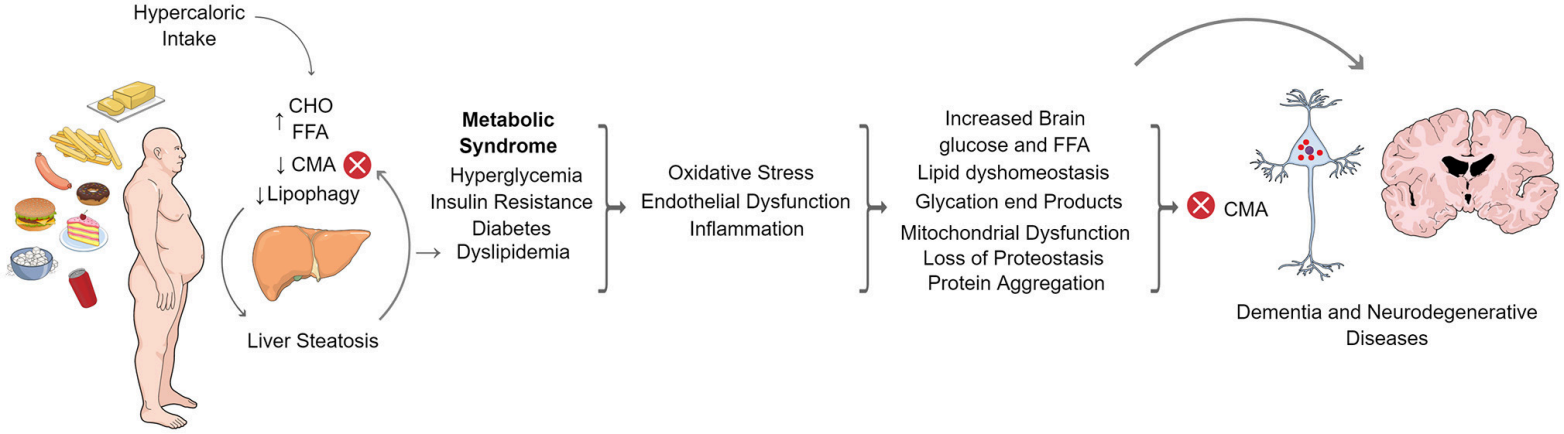
CMA in the crosstalk of metabolic dysfunction and neurodegenerative diseases. Increased caloric intake in humans produces elevated circulating levels of carbohydrates (CHO) and free fatty acids (FFA). In the liver, FFAs accumulate in lipid droplets leading to hepatic steatosis. FFAs also inhibit CMA and lipophagy. The latter contributes to hepatic fat accumulation and systemic dyslipidemia that, together with increased levels of glucose, leads to metabolic syndrome characterized by insulin resistance and diabetes. Metabolic syndrome is a general status also characterized by oxidative stress, inflammation and endothelial dysfunction. This condition is present throughout the body and is also capable of affecting the nervous system, resulting in lipid dyshomeostasis, mitochondrial dysfunction, loss of proteostasis and protein aggregation in the brain. All of these processes have been shown to alter proteostatic mechanisms, including CMA. Loss of neuronal proteostasis finally alters neuronal function which leads to dementia or the development of neurodegenerative diseases.

As part of the autophagic pathways, CMA has been involved in regulating the metabolism of different proteins. Many of these proteins are strongly associated with neurodegenerative diseases affecting humans, while others are involved in the regulation of metabolic pathways such as lipids and carbohydrate metabolism. In addition, neurodegenerative diseases and metabolic disorders are common features of aged humans and many reports associate the dysregulation in lipids or carbohydrate metabolism, with the risk of developing some type of neurodegenerative disease. It has also been reported that a high fat, or high carbohydrate diet (or glucose reach diet), can also increase neurodegeneration prevalence or progression. On the other hand, CMA activity, has been observed to decline with age and with a non-balanced dietary intake such as a high fat diet. Overall, CMA can be considered a common factor in the regulation of metabolic and neurodegenerative pathways and a dysregulation in CMA, provoked by normal aging, or by metabolic disorders induced by deficient nutritional habits, which could tilt the balance toward a pathological situation. Figure 3 shows an overview of how CMA could be connected with metabolic dysfunctions and neurodegenerative disorders.

## Author Contributions

AA and MB wrote the article. IEA wrote the article and created the figures. AM, JM, KC, and AC made important contributions, participated in discussions and provided corrections to the manuscript.

### Conflict of Interest Statement

The authors declare that the research was conducted in the absence of any commercial or financial relationships that could be construed as a potential conflict of interest.
